# Prognostic value of preoperative C-reactive protein to albumin ratio in patients with thymic epithelial tumors: a retrospective study

**DOI:** 10.1186/s12885-022-10234-x

**Published:** 2022-11-17

**Authors:** Yang-Yu Huang, Xuan Liu, Shen-Hua Liang, Yu Hu, Guo-Wei Ma

**Affiliations:** 1 State Key Laboratory of Oncology in South China, Sun Yat-Sen University Cancer Center, Collaborative Innovation Center for Cancer Medicine, Guangzhou, China; 2 Faculty of Biology, Medicine and Health, School of Biological Sciences, The University of Manchester, Manchester, UK

**Keywords:** Thymic epithelial tumors, Prognostic factor, C-reactive protein to albumin ratio, Overall survival, Recurrence free survival

## Abstract

**Background:**

The C-reactive protein to albumin ratio (CAR) is associated with poor prognosis in various cancers. However, its value in thymic epithelial tumors remains to be elucidated, we aimed to evaluate the prognostic significance of preoperative CAR in patients with surgically resected thymic epithelial tumors (TETs).

**Methods:**

We retrospectively collected data from 125 patients with TETs who underwent thymoma resection at our center. The best cutoff values ​​for the continuous variable, CAR, were obtained using X-tile software. Univariate and multivariate Cox regression analyses were used to evaluate CAR as an independent predictor of overall survival (OS) and recurrence-free survival (RFS). Kaplan–Meier analysis and log-rank tests were used to present risk stratification of patients based on CAR and the Glasgow-prognostic-score (GPS). The prognostic effect of CAR was assessed using a receiver operating characteristic curve.

**Results:**

Patients were categorized into high (≥ 0.17) and low (< 0.17) CAR groups according to the optimal cutoff value of 0.17. Univariate and multivariate analyses showed that CAR was an independent predictor of prognosis. World health organization stage, CAR level, GPS score, and drinking history were important independent prognostic factors for OS (*p* < 0.05). T stage, CAR level, and drinking history were important independent prognostic factors for RFS (*p* < 0.05). The area under the curve value of CAR to predict prognosis was 0.734 for OS and 0.680 for RFS.

**Conclusions:**

Elevated preoperative CAR was independently associated with poor OS and RFS after thymectomy. Therefore, CAR may be a valuable biomarker for the postoperative prognosis of TETs.

**Supplementary Information:**

The online version contains supplementary material available at 10.1186/s12885-022-10234-x.

## Introduction

Thymic epithelial tumors (TETs) are mediastinal tumors originating from epithelial cells of the thymus [1, 2]. They can be grouped into thymoma, thymic carcinoma, and thymic neuroendocrine tumors based on their histology. Although TETs represent the most common of the anterior mediastinum tumors in adults, their incidence is only 0.13 per 100,000 person-years, according to the American Cancer Registry [3]. In the world health organization (WHO) classification system, TETs are categorized into A, AB, B1, B2, B3, and rarer subtypes [4]. Surgical treatment is still the standard treatment for thymic epithelial tumors [5–8], and prognostic factors for postoperative survival include tumor size [9, 10], age [11], T stage [12], WHO histological classification [13], tumor vascular invasion [14], completeness of resection [15], and postoperative adjuvant therapy [16]. However, biomarkers with prognostic value have not yet been discovered and confirmed for this type of malignancy.

A growing number of reports indicate that both a systemic inflammatory response and nutritional status are important factors associated with long-term survival outcomes in various malignant tumors. Although the C-reactive protein to albumin ratio (CAR) has been repeatedly reported to be related to poor prognosis in various cancers, including pancreatic [17, 18], gastric [19], lung [20], and esophageal cancer [21], its role in TETs remains to be elucidated.

Given the rarity of TETs, few studies have investigated the preoperative CAR in patients with this tumor. Although thymoma is usually indolent, it is malignant. Therefore, we aimed to evaluate the prognostic value of CAR in patients with TETs. We assessed the relationship of CAR levels with overall survival (OS) and recurrence-free survival (RFS), as well as the predictive effects of CAR and the Glasgow-Prognostic-score (GPS).

## Materials and methods

This study was approved by the Medical Ethics Committee of Sun Yat-Sen University Cancer Center (B2020-353–01) and complied with the Declaration of Helsinki. Data were recorded at the Sun Yat-sen University Cancer Center, under the record number: RDDA2021002090.

We retrospectively collected the medical records of patients with TETs, who underwent thymoma resection at Sun Yat-sen University Cancer Center between May 2004 and August 2015. The patient inclusion criteria were as follows: (1) patients older than 18 years; (2) presence of histopathologically confirmed TET, including thymoma and thymic carcinoma (TC); (3) complete surgical resection (R0, no residual disease); and (4) complete relevant laboratory tests (routine blood and routine biochemical) within 7 days before surgery. Patients were excluded if: (1) radiotherapy or chemotherapy administered prior to surgery, before and after surgery, or an unknown sequence of treatment with surgery. (2) follow-up time of less than five years. (3) patients with more than one malignancy or history of other malignancies. (4) postoperative survival time of less than 3 months. (5) the patient only underwent thymoma biopsy. (6) the surgical method was cryoablation. (7) incomplete follow-up information.

### Data collection

Data were collected on the following clinical variables: hematological parameters (obtained within 1 week before surgery), albumin level (ALB), C-reactive protein level (CRP), patient's age, sex, drinking history (drinking alcohol every day, although the specific amount of drinking was not limited or described), smoking history, family history of tumor, histological subtype, tumor size, T stage, myasthenia gravis symptoms, and other clinical information.

### Follow-up

The follow-up strategy was every 6–12 months for the first two years, every 12 months for the third to fifth years, and then once annually. The follow-up included chest CT plain scan, hematological examination (routine blood, routine biochemical, tumor markers, etc.). The last follow-up was in August 2020. The primary endpoints were the OS and RFS.

### Variable definitions

All hematological parameters were collected within 7 days before surgery. Nutritional indicators were calculated as follows: CAR (C-reactive protein/albumin ratio); Glasgow-Prognostic-score (GPS) including CRP and albumin serum levels. CRP > 1 mg/dL was attributed 1 point; otherwise, 0 points were given. Albumin < 35 g/L was attributed 1 point; otherwise, 0 points were given.

### Data analysis

In this study, X-tile software was used to obtain the optimal CAR cutoff value (http://www.tissuearray.org/rimmlab). Statistical analyses were performed using SPSS 25.0 (IBM,Chicago, Illinois, USA) and R software (version 4.0.3; https://www.r-project.org/). Univariate and multivariate analyses were performed using Cox proportional hazards regression models, using hazard ratios (HRs) and 95% confidence intervals (CIs) to assess relative risk. Survival analysis was performed using the Kaplan–Meier method, and differences in survival were compared using the log-rank test. Receiver operating characteristic (ROC) curve analysis was performed to analyze the area under the ROC curve (AUC); all tests were two-way, and the significance level was set at *p* < 0.05.

## Result

### Patient characteristics

A total of 125 patients with TETs, including 64 men and 61 women, were included in this study. They exhibited an average age of 50.63 ± 12.63 years and an average tumor size of 6.77 ± 3.27 cm. In addition, Table 1 also includes patients with WHO staging, T staging, smoking history, drinking history, myasthenia gravis, and other relevant clinical information.

**Table 1:** Basic demographic data, disease specific characteristics (*n* = 125)

Characteristic	N	%
Gender		
Male	64	51.2
Female	61	48.8
Age(years)		
≤ 60	98	78.4
> 60	27	21.6
Smoking history		
Never	94	75.2
Ever	31	24.8
Drinking history		
No	109	87.2
Yes	16	12.8
Family history of tumor		
No	103	82.4
Yes	22	17.6
Tumor size(cm)		
≤ 6	70	56.0
> 6	55	44.0
pT stage		
T1	101	80.8
T2-3	24	19.2
WHO stage		
A-AB	45	36.0
B1-B3	69	55.2
C	11	8.8
Myasthenia gravis,		
No	115	92.0
Yes	10	8.0
CAR		
≤ 0.17	110	88.0
> 0.17	15	12.0
GPS		
0	114	91.2
1	11	8.8

*CAR* C-reactive protein/albumin ratio, *GPS* Glasgow-Prognostic-Score, *pT stage* Pathological T stage

### Optimal cut-off for preoperative CAR

Taking OS as the endpoint, the optimal cut-off value of preoperative CAR was determined to be 0.17 (*p* < 0.01) using X-Tile software. For further analysis, patients were assigned into high or low CAR groups (> 0.17 or ≤ 0.17, respectively).

### Association of CAR and GPS with survival outcomes

**Fig. 1:**
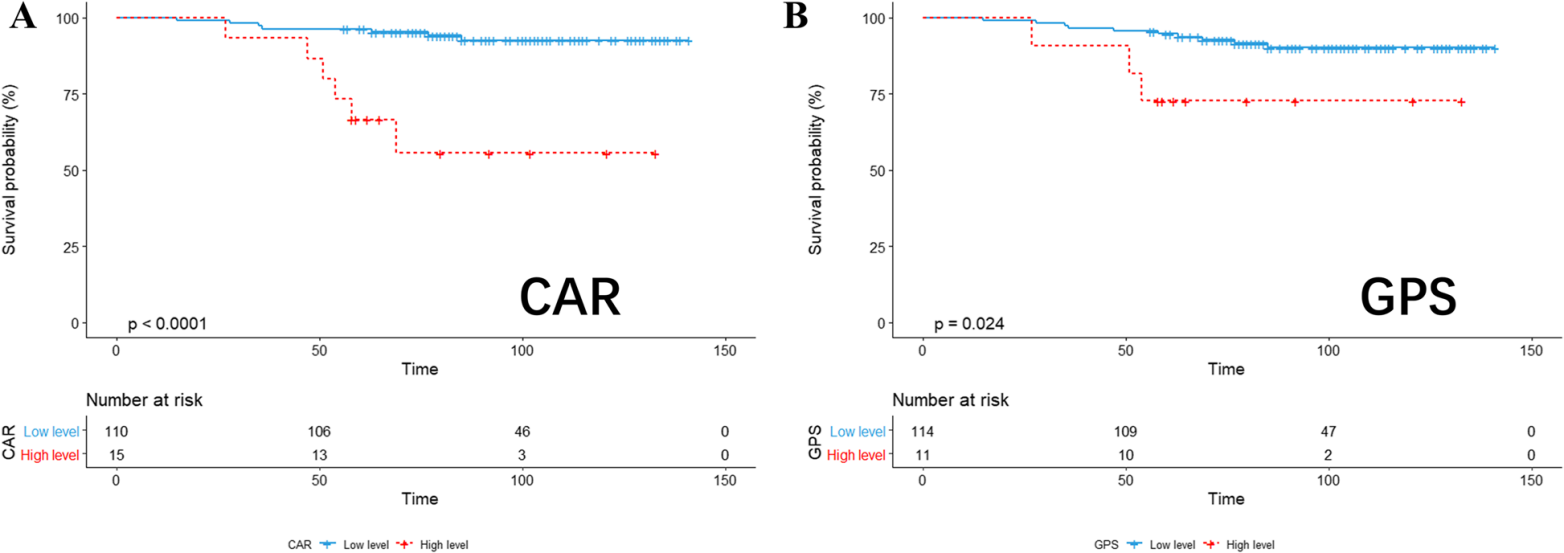
KM analysis of CAR (**A**) and GPS (**B**) based on overall survival

**Fig. 2:**
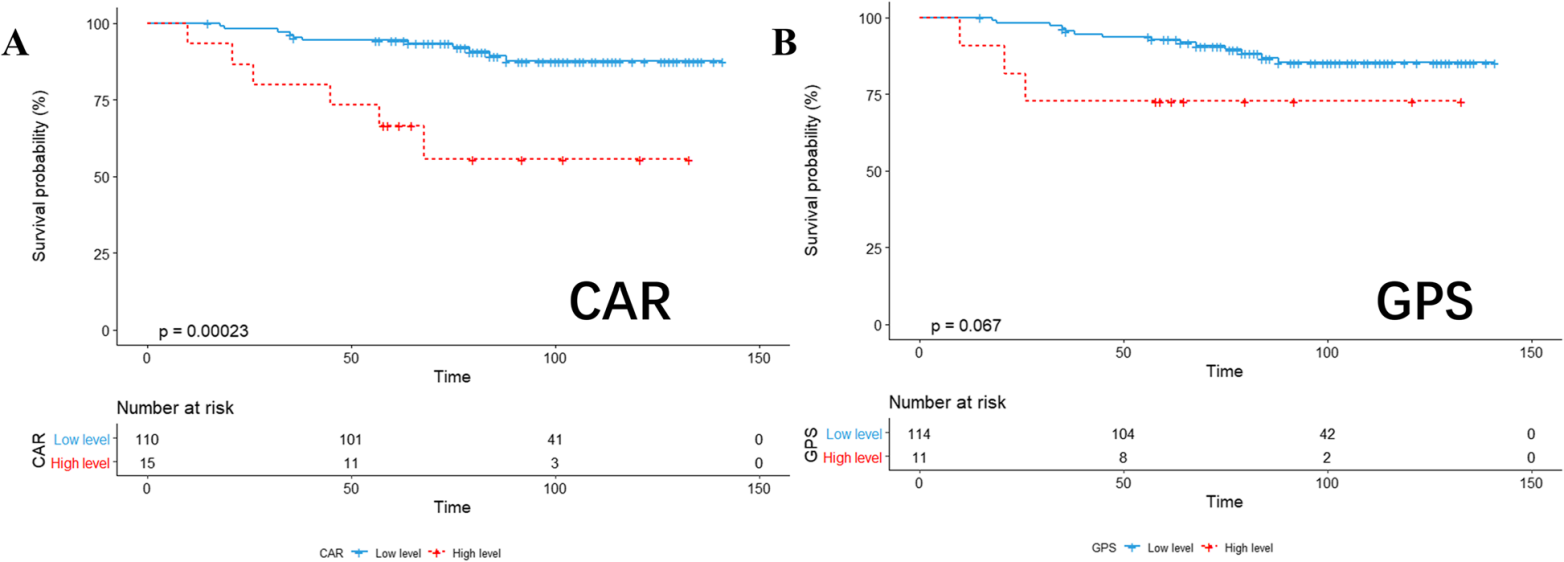
KM analysis of CAR (**A**) and GPS (**B**) based on relapse-free survival

**Fig. 3:**
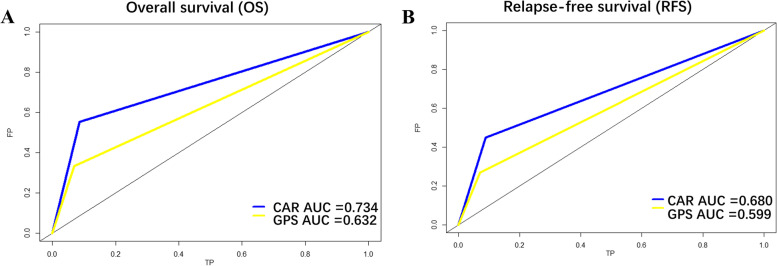
Receiver operating characteristic curve analysis for the sensitivity and specificity of the CAR and GPS based on overall survival (**A**) and relapse survival (**B**)

Taking OS and RFS as endpoints, we compared the OS and RFS results of patients assigned to the low-level and high-level CAR and GPS groups, and displayed them by Kaplan–Meier survival curves (Figs. 1 and 2). Finally, the receiver operating characteristic (ROC) curve was used to compare the AUC values ​​of the two groups (Fig. 3).

### Univariate and multivariate survival analysis based on overall survival

According to the results of the univariate Cox regression analysis, five variables were significantly associated with OS: WHO stage, T stage, drinking history, CAR, and GPS (Table 2). In multivariate Cox regression analysis, four parameters were defined as independent prognostic factors for OS: WHO stage (A-AB vs. B1-B3, HR = 0.489, at a 95% CI [range: 0.109–2.185] and A-AB vs. C, HR = 4.052, at a 95% CI [range: 0.918–17.886]), drinking history (HR = 6.362, at a 95% CI [range: 1.763–22.955]), CAR (HR = 27.091, at a 95% CI [range: 5.306–138.317]) and GPS (HR = 0.115, at a 95% CI [range: 0.017-0.770]) (Table 2).

**Table 2:** Univariate and multivariate analysis results in thymic epithelial tumor based on overall survival (*n* = 125)

Variable	Univariate analysis	Multivariate analysis		
	P	HR	95%CI	P
Gender				
Male vs Female	.191			
Age(years)				
≤ 60 vs > 60	.404			
Smoking history				
Never vs Ever	.257			
Drinking history	.006	Reference		
No vs Yes		6.362	1.763–22.955	.005
Family history of tumor				
No vs Yes	.295			
Tumor size				
≤ 6 vs > 6	.065			
pT stage				
T1 vs T2-3	.003			
WHO stage	.027	Reference		
A-AB vs B1-B3		.489	.109–2.185	.017
A-AB vs C		4.052	.918–17.886	
Myasthenia gravis,				
No vs Yes	.455			
CAR	.000	Reference		
≤ 0.17 vs > 0.17		27.091	5.306–138.317	.000
GPS		Reference		
0 vs 1	.037	.115	.017-.770	.026

*CAR* C-reactive protein/albumin ratio, *GPS* Glasgow-Prognostic-Score, *pT stage* Pathological T stage

### Univariate and multivariate survival analysis based on relapse-free survival

According to the results of the univariate Cox regression analysis, four variables were significantly associated with RFS: WHO stage, T stage, drinking history, and CAR (Table 3). In multivariate Cox regression analysis, three parameters were defined as independent prognostic factors for RFS: T stage (T1 vs. T2-3, hazard ratio, HR = 6.992, at a 95% CI [range: 2.585–18.908]), drinking history (HR = 5.549, at a 95% CI [range: 1.833–16.797]), and CAR (HR = 5.930, at a 95% CI [range: 2.019–17.418]) (Table 3).

**Table 3:** Univariate and multivariate analysis results in thymic epithelial tumor based on relapse-free survival (*n* = 125)

Variable	Univariate analysis	Multivariate analysis		
	P	HR	95%CI	P
Gender				
Male vs Female	.858			
Age(years)				
≤ 60 vs > 60	.348			
Smoking history				
Never vs Ever	.585			
Drinking history	.013	Reference		
No vs Yes		5.549	1.833–16.797	.002
Family history of tumor				
No vs Yes	.922			
Tumor size				
≤ 6 vs > 6	.070			
pT stage	.000	Reference		
T1 vs T2-3		6.992	2.585–18.908	.000
WHO stage				
A-AB vs B1-B3	.016			
A-AB vs C				
Myasthenia gravis,				
No vs Yes	.375			
CAR	.001	Reference		
≤ 0.17 vs > 0.17		5.930	2.019–17.418	.001
GPS				
0 vs 1	.081			

*CAR* C-reactive protein/albumin ratio, *GPS* Glasgow-Prognostic-Score, *pT stage* Pathological T stage

## Discussion

In this study, we show that a high preoperative CAR is associated with poor patient outcomes after thymectomy, including overall survival and recurrence-free survival. Additionally, we confirm that GPS is an independent prognostic factor for overall survival in patients with TETs.

Consistent with previous findings, we show that T stage and WHO stage are independently associated with the postoperative prognosis of TETs [12, 22, 23]. Interestingly, our multivariate Cox analysis found that a history of drinking was independently associated with poor OS and RFS. Indeed, in a multicenter case–control study, Sabroe et al. found that alcohol consumption is a risk factor for thymoma [24]. However, its relationship with postoperative prognosis of patients is unclear, and further research is therefore needed.

CRP is an important acute-phase reactive protein that is predominantly produced by hepatocytes, and its level increases with the inflammatory response [25]. In 2017, Moser et al. found that serum CRP levels help to indicate highly aggressive TETs and may be an indicator of tumor recurrence during patient follow-up [26]. In 2019, Moser et al. found that the CRP-fibrinogen score (CFS) was an independent predictor of recurrence in patients with TETs [27].

Albumin is also generated by the liver and maintain intravascular osmotic pressure and promote substance transport. At the same time, albumin is an important nutrient, reflecting the nutritional status of the human body. Additionally, hypoalbuminemia is associated with a persistent systemic inflammatory response [28]. Multiple studies have shown that the development of hypoalbuminemia is secondary to elevated serum CRP levels [29, 30]. Malnutrition is related to a poor prognosis in patients with thymic epithelial tumors [31]. Furthermore, Ma et al. reported that preoperative albumin levels were associated with postoperative RFS in TET patients [32].

The impact of systemic inflammatory responses on the short- and long-term outcomes of various tumors has been widely reported [33–35]. Among them, the prognostic value of a neutrophil count, a neutrophil-to-lymphocyte ratio (NLR), and a platelet-lymphocyte ratio (PLR) in TETs has been reported and confirmed [36, 37]. The association between albumin and nutritional status reflects the systemic inflammatory response and nutritional status of patients and has been confirmed [38]. In addition, Okamoto et al. indicated that CAR may be the most useful prognostic indicator in postoperative immuno-nutritional parameters of non-small cell lung cancer [39]. Due to the low incidence of thymoma, the current research on these potentially effective prognostic markers is relatively limited. In our study, CAR showed the greatest discriminative power, which was shown to be independently associated with OS and RFS. By comparing the area under the ROC curve (AUC), it can be seen that the prognostic prediction ability of CAR is superior to that of the GPS.

This study includes some limitations: First, it was performed at a single institution using a retrospective design and relatively few patients. It is therefore necessary to conduct a prospective multicenter study to test our findings in a larger patient cohort. When the model is applied to an external database, its validity needs to be verified. Additionally, only patients with primary TET surgery were included in this study; therefore, our findings may not be suitable for prognosis prediction in patients with recurrent TETs. and then, the threshold of the indicator CAR in this study was calculated based on the patients in this study, and a more precise threshold may be obtained with a larger sample size in the future. Finally, we were unable to investigate the impact of postoperative CAR dynamics in patients with thymoma.

## Conclusions

Through retrospective analysis of more than 10 years of patient data, Our findings suggest that preoperative serum CAR level is an independent prognostic factor for OS and RFS. Therefore, CAR appears to be a powerful biomarker for the postoperative prognosis of TETs, but further prospective and multicenter studies are needed to confirm our results.

## Supplementary Information

**Additional file 1: Figure 1.** KM analysis of T stage (A)、WHO（B） and Drinking history (C) based on overall survival.

**Additional file 2: Figure 2.** KM analysis of T stage (A)、WHO（B） and Drinking history (C) based on relapse-free survival.

**Additional file 3: Figure 3.** Nomogram predicting 3- ,5- and 10- overall survival after thymectomy for thymic epithelial tumors patients.

## Data Availability

If the investigator is interested in the clinic data, all clinicopathological information about our patient can be obtained by contacting the corresponding author, We have uploaded the data of this study in the Research Data Deposit of SYSUCC, and its number was RDDA2021002090 (http://www.resea rchdata.org.cn/).

## Abbreviations

CAR: C-reactive protein/albumin ratio
GPS: Glasgow-Prognostic-Score
pT stage: Pathological T stage NLR:neutrophil-to-lymphocyte ratio
ALB: Albumin
PLR: Platelet-lymphocyte ratio
pT stage: Pathological T stage
OS: Overall survival
RFS: Recurrence-free survival
TC: Thymic carcinoma
HR: Hazard ratio
CI: Confidence interval
ROC: Receiver operating characteristic curve
AUC: Area under the curve
TET: Thymic epithelial tumor
