# Supplementary figures and images for: Prognostic value of preoperative C-reactive protein to albumin ratio in patients with thymic epithelial tumors: a retrospective study
Source: BMC Cancer. 2022 Nov 17;22:1183. doi: 10.1186/s12885-022-10234-x (PMC9670652; doi:10.1186/s12885-022-10234-x)

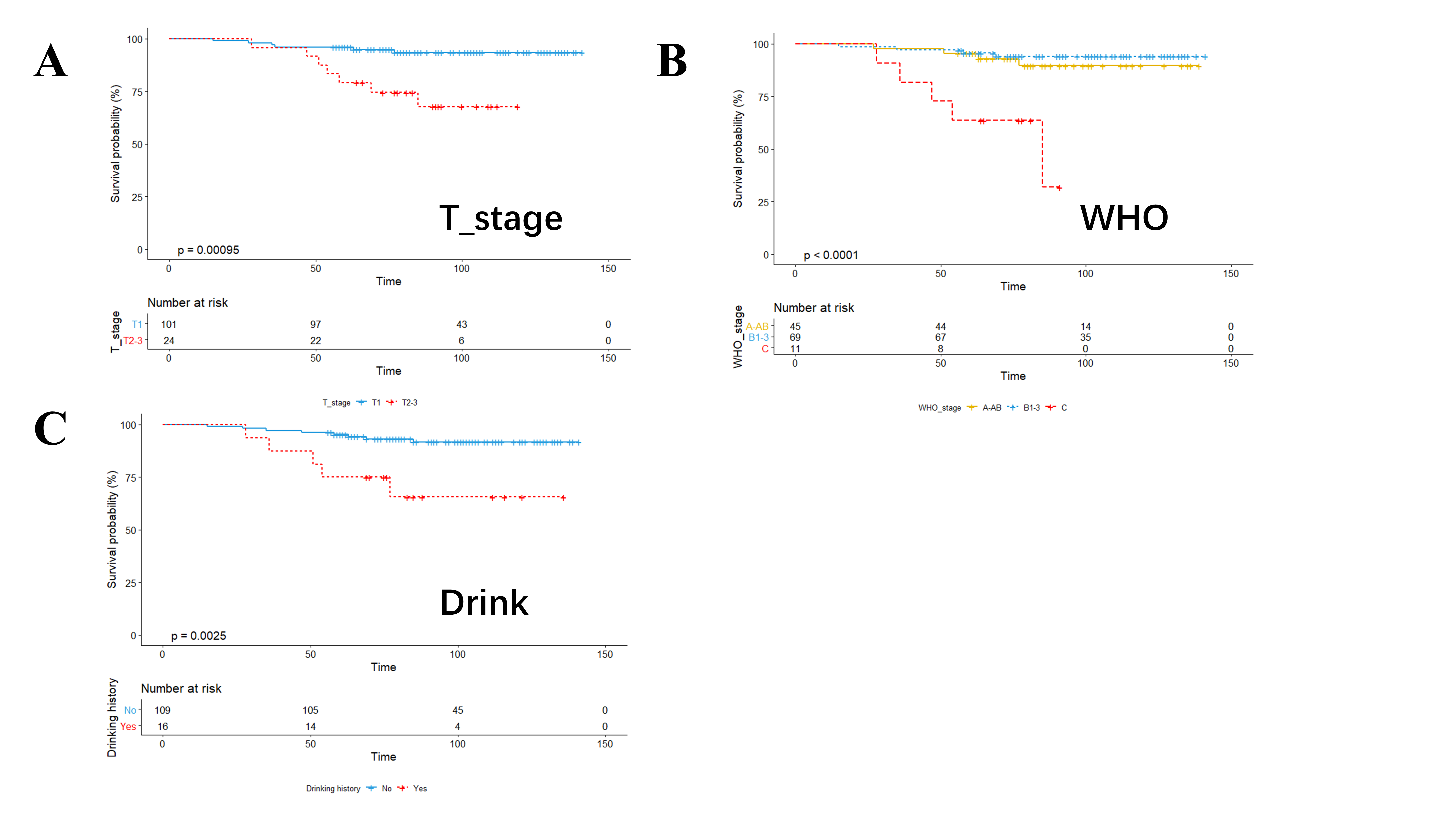

Supplement: Supplementary file 1 — Additional file 1: Figure 1. KM analysis of T stage (A)、WHO（B） and Drinking history (C) based on overall survival. [file 12885_2022_10234_MOESM1_ESM.tif]

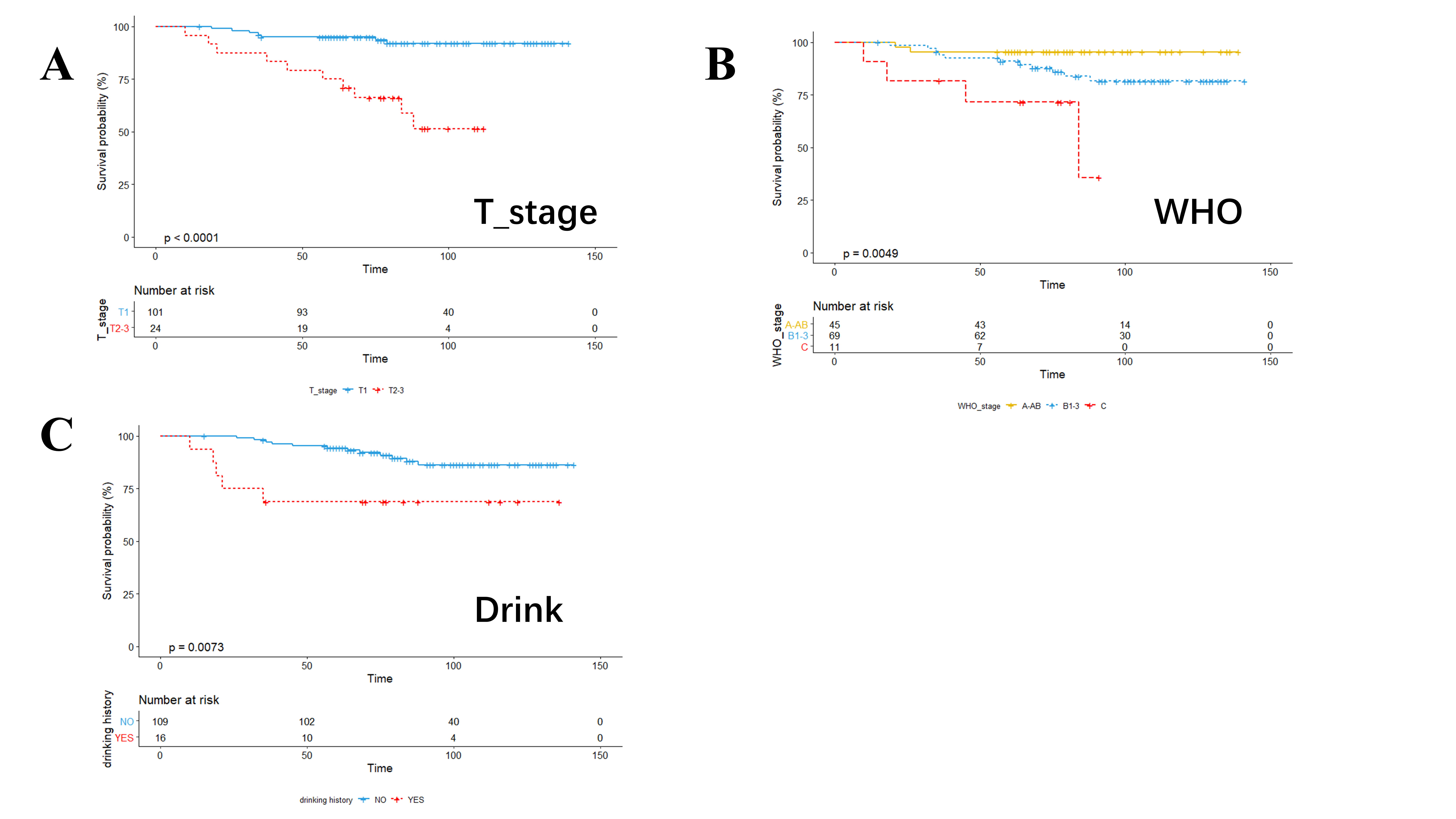

Supplement: Supplementary file 2 — Additional file 2: Figure 2. KM analysis of T stage (A)、WHO（B） and Drinking history (C) based on relapse-free survival. [file 12885_2022_10234_MOESM2_ESM.tif]

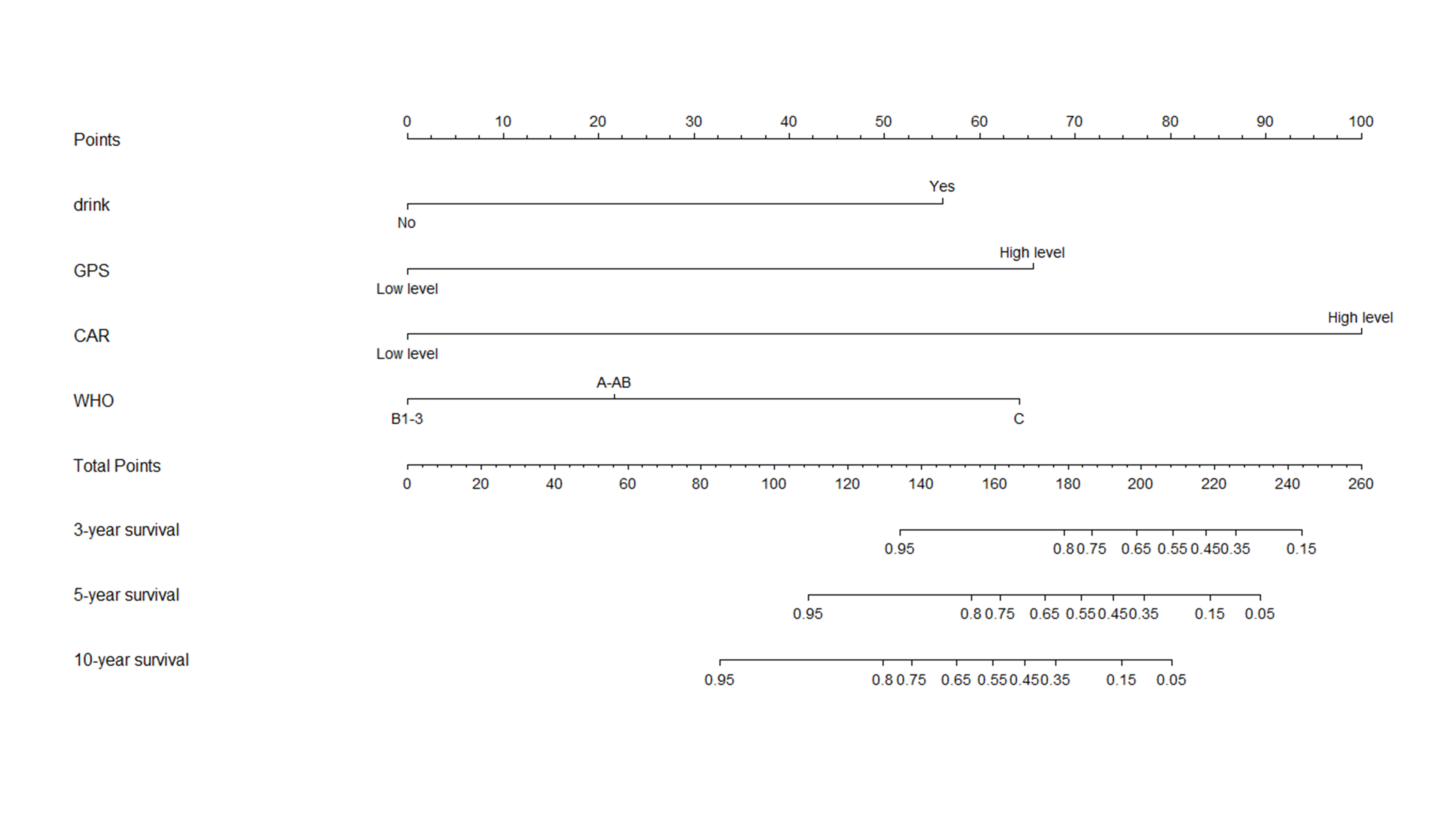

Supplement: Supplementary file 3 — Additional file 3: Figure 3. Nomogram predicting 3- ,5- and 10- overall survival after thymectomy for thymic epithelial tumors patients. [file 12885_2022_10234_MOESM3_ESM.tif]
